# First study on molecular epidemiology of dermatophytosis in cats, dogs, and their companions in the Kurdistan region of Iraq

**DOI:** 10.14202/vetworld.2022.2971-2978

**Published:** 2022-12-29

**Authors:** Karwan Idrees Jarjees, Nawzat Aboziad Issa

**Affiliations:** 1 Department of Pathology and Microbiology, College of Veterinary Medicine, University of Duhok, Kurdistan Region, Iraq; 2 Department of Surgery and Internal Medicine, College of Veterinary Medicine, University of Duhok, Kurdistan Region, Iraq

**Keywords:** cats, coronavirus disease 2019, dermatophytosis, dogs and human, Kurdistan region-Iraq, molecular identification

## Abstract

**Background and Aim:**

Dermatophytosis is a zoonotic infection of the hair, skin, or nails in animals and humans caused by dermatophytes fungi. This study aimed to estimate the prevalence of dermatophytosis and its associated factors in cats, dogs, and humans in the Kurdistan region of Iraq.

**Materials and Methods:**

Skin scraping samples were taken from cats, dogs, and humans with or without skin lesions. In total, 271 samples were collected; 133 from cats, 94 from dogs, and 44 from humans. The collected samples were cultured on dermatophyte test media for fungal isolation and molecular identification.

**Results:**

The prevalence of the disease was 44.36%, 40.43%, and 65.91% in cats, dogs, and humans, respectively. *Microsporum canis*, the most frequently isolated dermatophyte, occurred in 94.92% of cats, 92.11% of dogs, and 100.0% of humans whereas, *Trichophyton mentagrophytes* was only isolated from 5.08% of cats to 7.89% of dogs. Animals and humans at younger ages were more susceptible to the infection. Males were more susceptible than females among animals, while the reverse was true in humans. Housed cats were at higher risk of dermatophytosis than outdoor-reared cats, whereas outdoor-reared dogs were at higher risk of dermatophytosis than indoor-reared dogs. The affected skin in animals and humans is significantly associated with higher prevalence rates of the disease. Contact with infected cats and dogs was associated with increased infection rates in humans. Patients with a history of coronavirus disease 2019 (COVID-19) were found to be at higher risk of dermatophytosis than those with no history of COVID-19.

**Conclusion:**

Awareness should be raised among people about the zoonotic aspect of the disease, especially among those with COVID-19, to avoid contact with cats and dogs, who are at risk of the disease.

## Introduction

Dermatophytosis, a highly contagious and zoonotic fungal disease of the skin in animals and humans, is commonly caused by the dermatophyte fungal species of *Trichophyton, Microsporum*, and *Epidermophyton* [1]. Dermatophytosis is globally distributed, with increasing numbers of annual cases not only in humans but also in animals. The disease has gained special attention in public health, particularly in cats and dogs [2], as the most common source of human infection [3]. *Microsporum canis* and *Trichophyton* species are the most important species of dermatophytes isolated from infected dogs, cats, and their companions [1, 4].

Different diagnostic techniques and methods rely on the diagnosis and identification of the causative agents of dermatophytosis. Hitherto, the diagnosis was based on clinical signs, which are unreliable due to the variable nature of the dermatological findings and the similarity of other skin diseases that mimic the characteristic dermatophyte lesions [5]. Direct microscopic examination and the isolation of the dermatophytes on mycobiotic agars are considered the conventional gold standard diagnostic techniques; however, further post-culturing identification through biochemical tests or microculture could be required. Dermatophyte culture-based identification is laborious, time-consuming, and has poor sensitivity [6], while more rapid, accurate, reliable, sensitive, and specific diagnostic tools such as molecular diagnostic methods provide simple and precise tools for dermatophyte species characterization and phylogenetic analysis [5]. This study relied on molecular diagnostics for the identification and characterization of isolated dermatophyte species, and to the best of our knowledge, no study has been conducted so far to determine the prevalence of dermatophytosis among cats, dogs, and their companions in the Kurdistan region of Iraq. It was also unknown what risk factors were associated with the prevalence of the disease and to what extent such factors could play a role in the spreading of the disease in animals and humans.

Therefore, this study aimed to estimate the prevalence of dermatophytes among cats, dogs, and their companions, relying on molecular techniques for the identification and characterization of isolated dermatophytes, and to examine the role of risk factors and their association with the prevalence of the disease in both animals and humans.

## Materials and Methods

### Ethical approval

All ethical considerations were dully addressed, and the study was conducted with the approval of the Animal Ethical Committee at the College of Veterinary Medicine, University of Duhok, Iraq (Permit number: CVM202174UoD).

### Study period and location

This study was conducted from June 2021 to July 2022 in the Kurdistan region (Duhok, Erbil, and Sulaimaniya Governorates) of Iraq.

### Sampling and samples collection

A total of 271 samples were collected from cats suspected of having dermatophytosis (n = 133), dogs (n = 94) referred to local veterinary clinics or from animal shelters, and people (n = 44) who were in contact with these animals. Sample collection was based on clinical manifestations and a Wood’s lamp examination. During sample collection, age, sex, clinical observation, and predisposing factors were recorded using a questionnaire. Before collecting samples, the infected area was cleaned with 70% (v/v) ethanol. Skin scrapes and hair-derived tissues from cats and dogs, from advancing lesion margins, and from fluorescence identification were collected by a veterinarian aseptically using sterile blades and forceps and transferred into sterile plastic Petri dishes. Among people, emphasis was placed on information regarding contact with infected cats and dogs, and other information, such as the sex and age of infected patients was recorded. Besides, coronavirus disease 2019 (COVID-19) infection (samples collected 2 months post-infection). All sample collections were performed by a dermatologist, strictly adhering to the instructions and directives of the related health department in terms of COVID-19 and the use of personal protective equipment.

### Culture and microscopic examination

Collected samples were cultured on dermatophyte test media (DTM, HiMedia, India, REF: M188-500G) agar plates containing papaic digest of soybean meal (10 g/L), glucose (10 g/L), phenol red (0.2 g/L), agar (20 g/L) with yeast extract (4 g/L), and gentamicin (50 mg/L) with cycloheximide (500 mg/L) (HiMedia Company, India) at a final pH of 5.6 ± 0.2 prepared per the manufacturer’s instructions. All inoculated plates were then incubated at 30°C for 4 weeks. Plates were examined twice a week for any fungal growth, and in the absence of growth during week 4, these samples were considered negative.

Cultures of the isolated dermatophytes were initially identified by examining their colony morphologies (macroscopically) on DTM and microscopic characteristics. Dermatophytes were identified based on the change in DTM color from yellow to red as dermatophytes produce alkali substances that promote the pH and change the medium’s phenol red from yellow to red. For the macroscopic identification, the growth texture, rate, and pigmentation of the reverse and front sides of the culture were employed. Microscopically, the isolates were identified using adhesive tape and lactophenol cotton blue stain (LPCB). The sticky piece of tape with fungal structures adhered to the slide with a layer of LPCB.

### Molecular identification of the isolates

DNA was extracted from positive dermatophytes’ cultures using a fungal DNA preparation kit (Jena Bioscience, Germany). Two sets of primers were used, Mc-VelB-F (5′-CTTCCCCACCCGCAACATC-3′) and Mc-VelB-R (5′-TGTGGCTGCACCTGAGAGT GG-3′), for *M. canis* [7]. Whereas, CHS1 gene primers, the forward CHS1 1S (5′-CAT CGA GTA CAT GTG CTC GC-3′) and the reverse CHS1 1R (5′-CTC GAG GTC AAA AGC ACG CC-3′), were used for *Trichophyton mentagrophytes* [8]. Primers’ sets were supplied by (Macrogen, Inc. South Korean public, Biotechnology Company. H000126660).

Polymerase chain reaction (PCR) amplification was performed using a Thermal Cycler (GeneAmp* PCR system 9700 Applied Biosystems, USA) in a total volume of 20 μL. For Mc-VelB, the PCR mixture consisted of 1.4 μL DNA, 10 μL master mix (ADDBIO Inc. Taq master REF 36101, lot 21010), 0.8 μL of each primer, and 7 μL of nuclease-free distilled water. The following conditions were applied when running PCR for Mc-VelB: The initial denaturation was performed at 94°C for 4 min, and then 35 PCR cycles were performed as follows: 94°C for 1 min, 62°C for 1 min, and 72°C for 1 min, and the terminal extension step was 72°C for 7 min.

PCR for CHS1 in a 20 μL reaction mixture volume consisted of 10 μL of master mix, 1 μL of each forward and reverse primer, 1.5 μL of the genomic DNA template, and 6.5 μL of nuclease-free distilled water. The conditions used were an initial denaturation at 94°C for 5 min, then 35 cycles of denaturation at 94°C for 1 min, an annealing step at 63°C for 2 min, and an extension step 68°C for 2 min, and the 7 min terminal extension was set at 68°C [8].

PCR products were analyzed with ethidium bromide staining of a 1% agarose gel electrophoresis alongside DNA ladder (100 bp) and then visualized using UV light. In total, 10 μL of the amplified products were electrophoresed on the gel, the produced bands were visualized under UV light, and amplicon of 200 bp and 450 bp was considered positive for *M. canis* and *T. mentagrophytes*, respectively.

For sequencing, four representative positive PCR samples (three of *M. canis* and one of *T. mentagrophytes*) were tested against universal primers internal transcribed spacer (ITS gene from Macrogen, South Korean public), the forward primer (ITS1 5′-TCCGTAGGTGAACCTGCGG-3′) and a reverse primer (ITS4 5′-TCCTCCGCTTATTGATATGC-3′) were used [9, 10]. The PCR cycling condition was chosen per the manufacturer’s instructions using 50 μL of the reaction mixture [10]. The PCR products were electrophoresed on 2% agarose gel with a 100 bp DNA ladder. The amplified 700 bp PCR products were sent to a commercial company for purification and sequencing (Macrogen Inc., South Korea).

To generate a phylogenetic tree, sequenced *M. canis* and *T. mentagrophytes*, which represent different locations of the study area, were calculated using a multiple sequence alignment in MEGA version 11 (https://www.megasoftware.net/show_eua). The obtained sequences were aligned together with sequence accession numbers taken from NCBI GenBank for comparisons using the Muscle algorithm. The phylogenetic tree was inferred using the Maximum Likelihood method based on the Tamura-Nei Model, with 1000 bootstrap replicates being performed to estimate the node reliability.

### Statistical analysis

GraphPad Prism software version 8.0.1 (https://www.graphpad.com/scientific-software/prism/) was used to perform the statistical analysis of the data, and the Chi-squared and Fisher’s exact tests were used to determine the statistical significance of the differences between the factors associated with the prevalence of dermatophytes in the examined cats, dogs, and humans. Differences were considered significant at p < 0.05. The obtained data were expressed quantitatively using percentages for the prevalence of the disease.

## Results

### Clinical findings of dermatophytosis in infected cats, dogs, and patients

**Figure-1:**
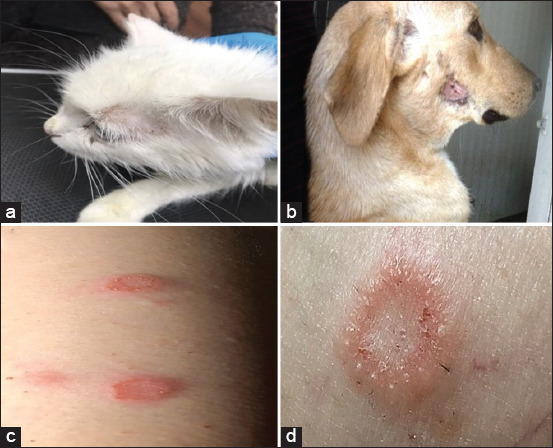
Clinical lesions of dermatophytosis in (a) cat, (b) dog, and (c and d) human.

Clinically, the infected animals showed signs of hair loss, skin crust, erythema, and pruritus in different body parts. In infected cats and dogs, the lesions were most common in the head, followed by the trunk, and less commonly found in the legs and tail; lesions were single and presented as multifocal alopecia, erythema, papules, pustules, scales, and crusts with a distinctive circular shape (ringworm). Whereas in examined patients, lesions were commonly observed in the hands (Figure-1).

**Figure-2:**
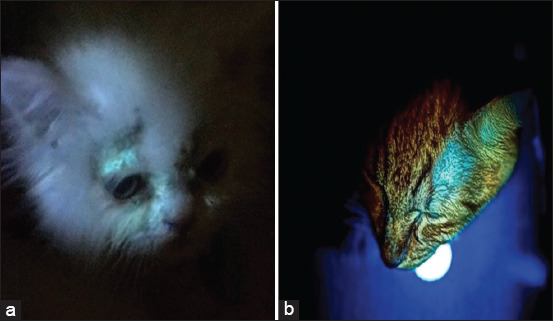
A positive Wood’s lamp test. (a) A bright green fluorescent spot was detected on the base of hair shaft over the eyelid in a long-haired cat with positive to *Microsporum canis*. (b) Green fluorescent spot was identified over the left eye of a short-haired cat with positive for *M. canis*.

Positive Wood’s lamp examination revealed an apple green fluorescence of individual hair follicles close to the base of the hair shaft (Figure-2).

### Isolation and molecular identification of dermatophytes from cats, dogs, and humans

**Figure-3:**
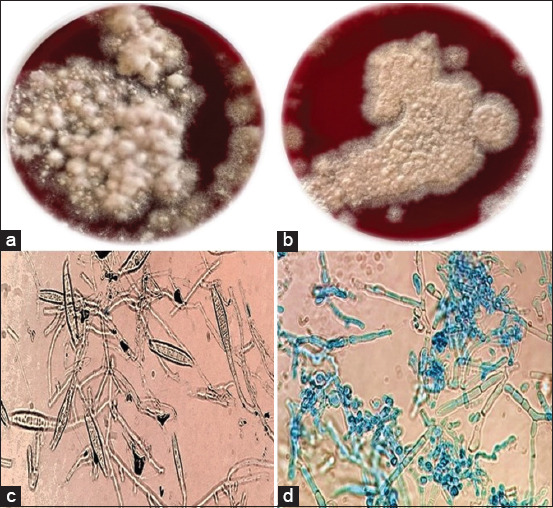
Isolated dermatophytes on dermatophyte test media after 17 days at 30°C. (a) Colony morphology of *Microsporum canis*, (b) colony morphology of *Trichophyton mentagrophytes*, (c) lactophenol cotton blue (LPCB) stained *M. canis* under a light microscope at a 40×, and (d) LPCB stained *T. mentagrophytes* under a light microscope at a 40×.

Collected samples were cultured on DTM agar plates (HiMedia), and morphological (macroscopic and microscopic) approaches were first applied for the identification of growth, followed by molecular identification. Macroscopically, *M. canis* presented white-to-pale yellow fungal colonies at the top of the plate, and *T. mentagrophytes* showed white-to-cream-colored powdery surface colonies on DTM (Figures-3a and b). Microscopically, LPCB-stained *M. canis* revealed the presence of septate hyphae with spindle-shaped macroconidia having an asymmetrical apical knob, and *T. mentagrophytes* revealed numerous single-celled microconidia that formed clusters of smooth-walled spheres with the presence of septate hyphae (Figures-3c and d).

The isolation rates of dermatophytes on DTM agar were 44.36% (59/133), 40.43% (38/94), and 65.91% (29/44) from cats, dogs, and humans, respectively (Table-1).

**Table-1:** Isolation rates of dermatophytes from skin scrapings and hairs of cats, dogs, and humans on DTM.

Samples source	Total			*Microsporum canis*		*Trichophyton mentagrophytes*	
		
	No. of examined	No. of positive	%	No. of positive	%	No. of positive	%
Cats	133	59	44.36	56	94.92	3	5.08
Dogs	94	38	40.43	35	92.11	3	7.89
Human	44	29	65.91	29	100.00	0	0.00
Total	271	126	46.49	120	95.24	6	4.76

DTM=Dermatophyte test media

**Figure-4:**
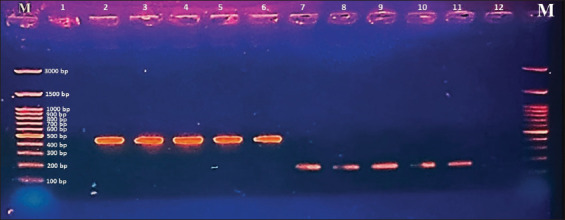
Amplification of Mc-VelB gene specific for *Microsporum canis* and CHSI gene specific for *Trichophyton mentagrophytes*. Lane 100 bp: DNA ladder. M: Molecular marker, lanes 1 and 12: Negative control with water, lines 2–6: Amplified CHSI (450 bp) gene in isolated *T. mentagrophytes*, and lines 7–11: Amplified Mc-VelB (200 bp) gene in *M. canis* isolates.

The isolates were identified and confirmed by PCR using the *Mc-VelB* gene specific for *M. canis* and the *CHS1* gene for *T. mentagrophytes*. Initially, the isolates were tested for the *Mc-VelB* gene, and negative samples were tested for the *CHSI* gene. Amplicons of 200 bp and 450 bp were considered positive for *M. canis* and *T. mentagrophytes*, respectively (Figure-4). The results showed that DNA fragments of 200 bp for the *Mc-VelB* gene of *M. canis* were successfully amplified at rates of 94.92%, 92.11%, and 100% in isolates from cats, dogs, and humans, respectively. Whereas, 450 bp for the *CHSI* gene of *T. mentagrophytes* was only identified in isolated dermatophytes from cats and dogs at rates of 5.08% and 7.89%, respectively.

**Figure-5:**
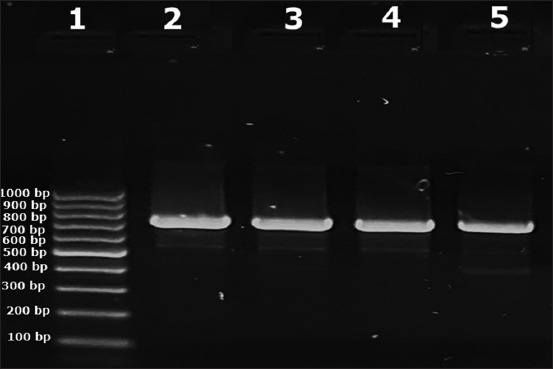
Polymerase chain reaction amplification of genomic DNA of *Microsporum canis* and *Trichophyton mentagrophytes* using ITS universal gene yielded (700 bp). Lane 1: 100 bp ladder DNA marker, lanes 2–4: *M*. *canis*, and Lane 5: *T. mentagrophytes*.

For sequencing, representative positive PCR products of three isolates of *M. canis* and one isolate of *T. mentagrophytes* were tested for the *ITS* universal gene, which was successfully amplified at 700 bp PCR product (Figure-5). BLAST analyses against globally published dermatophytes based on nucleotide sequence comparisons of partial *ITS* sequences revealed that sequences similarity ranged from 87.39% to 99.71% with *M. canis* and from 95.9% with *T. mentagrophytes*. The sequences were deposited in GenBank with accession numbers (ON209159, ON221378, and ON221386) for *M. canis* sequence and (ON221385) for *T. mentagrophytes*.

**Figure-6:**
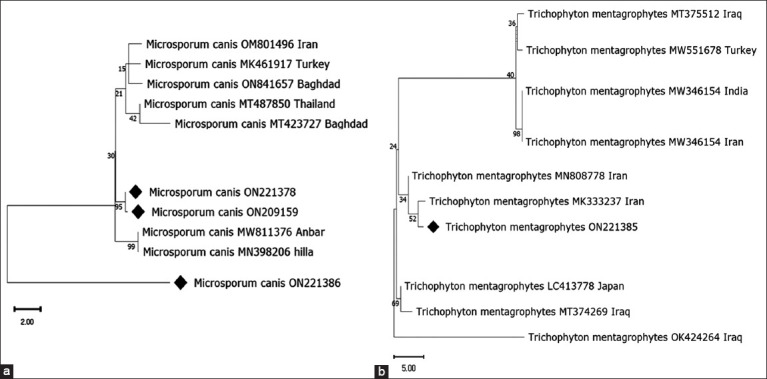
(a) Phylogenetic tree of *Microsporum canis* and (b) *Trichophyton mentagrophytes*: As interfered from of partial sequence; 5.8S ribosomal RNA gene. Black diamond refers to sequence obtained from the present study; others represent sequences from references.

A Maximum Likelihood phylogenetic tree of *M. canis* and *T. mentagrophytes* (Figure-6) sequences was constructed using MEGA 11. The bootstrap consensus tree of *M. canis* inferred from 1000 replicates with the highest log likelihood (−4002.46) and *T. mentagrophytes* (−3775.67). The percentage of trees in which the associated taxa clustered together is shown above the branches. The phylogenetic tree of *M. canis* (Figure-6a) shows that the sequences (ON209159 and ON221378) clustered with sequences previously detected from certain countries, for example, Iraq (MN398206, MW811376, MT423727, and ON841657), Turkey (MK461917), Iran (OM801496), and Thailand (MT487850); whereas, ON221386 sequences were divergent and not clustered with any of the used sequences.

The phylogenetic tree of *T. mentagrophytes* shows that the ON221385 clades were positioned near the sequences previously detected in Iran (MK333237 and MN808778), Japan (lc413778), and Iraq (MT374269). The isolate was clustered with sequences previously detected from Iraq, Iran, Turkey, and Iraq (Figure-6b).

### Factors associated with the prevalence of dermatophytosis among cats, dogs, and humans

Multivariable conditions associated with the prevalence of the disease among the examined animals and humans were considered and analyzed (Table-2). Data revealed that younger cats and dogs were more susceptible; the prevalence of the disease was 45.8% in cats and 45.6% in dogs aged below 1 year, compared to 38.5% and 32.4% in their counterparts aged 1–3 years. Concerning sex, the prevalence of the disease was higher in males than in females. In terms of residence, the prevalence was higher in household cats (46.8%) than in outdoor cats (31.8%). Whereas, the prevalence was higher among outdoor dogs (56.7%) than among indoor dogs (32.8%). With respect to skin conditions, the prevalence rates were significantly higher (p < 0.05) among cats and dogs with affected skin (62.1% and 62.5%, respectively) than among their counterparts with apparently healthy skin (26.9% and 17.4%, respectively).

**Table-2:** Risk factors associated with the prevalence of dermatophytes in examined dogs and cats.

Variable	Cats (n = 133)			Dogs (n = 94)		
	
	No. of examined	No. of positive	%	No. of examined	No. of positive	%
Age group						
1>year	107	49	45.8	57	26	45.6
1–3 years	26	10	38.5	37	12	32.4
Gender						
Males	63	30	47.6	47	19	40.4
Females	70	29	41.4	47	19	40.4
Residence						
Outdoor	22	7	31.8	30	17	56.7
Indoor	111	52	46.8	64	21	32.8
Skin condition						
Healthy	67	18	26.9	46	8	17.4
Dermatitis/affected skin	66	41	62.1*	48	30	62.5**

* Significant different at p *<* 0.05,

** Significant different at p *<* 0.01

Among the examined patients, the prevalence rates were affected by age, sex, skin condition, and contact with cats and dogs, in addition to the presence of COVID-19. Higher prevalence rates were found in younger people, females, patients with affected skin, and people who were in direct contact with pets (Table-3). The prevalence of the disease was higher (77.8%) in people with COVID-19 or those who had newly recovered from the disease than in non-infected people (47.8%).

**Table-3:** Risk factors associated with the prevalence of dermatophytes among the examined patients.

Variable	Human (n = 44)		

	No. of examined	No. of positive	%
Age group			
>18 years	34	19	55.88
<18 years	10	10	100.00
Gender			
Males	19	9	47.37
Females	25	20	80.00
Skin condition			
Healthy	14	1	7.14
Dermatitis	30	28	93.33**
Contact with infected animal			
Contact	26	22	84.62
Non-contact	18	7	38.89
COVID-19			
Infected	27	21	77.78
Non-infected	17	8	47.06

** Significant different at p *<* 0.01

## Discussion

Dermatophytosis is a ubiquitous and contagious superficial skin disease of humans and other animals, including cats and dogs. In animals, the disease is commonly caused by *M. canis* and *Trichophyton* species [11]. Clinically, the disease is characterized by alopecia, crusty lesions, erythema, and pruritus in various body parts. We found that lesions were mainly on the heads and trunks of infected cats and dogs and in the hands of infected people, which is in line with what has previously been reported [12–14]. The higher rates of *Tinea manuum* infection (lesions in hands) among patients are due to handling and direct contact with infected pet cats and dogs, which reflect inadequate sanitation and awareness among locals toward the disease.

For precise species-level identification of the isolated dermatophytes, molecular identification and DNA sequencing techniques were used to determine the prevalence rates due to the similarity of their phenotypic characteristics between typical and atypical strains of *M. canis* [15, 16]. In this study, the isolated rates of dermatophytes from cats, dogs, and humans were 44.36%, 40.43%, and 65.91%, respectively. In our study, *M. canis* was the dominant isolate (95.24%). *Microsporum canis*, a zoophilic dermatophyte, is a major cause of dermatophyte infections in humans and animals [4, 14]. *Trichophyton mentagrophytes* was isolated at lower rates and only from cats and dogs; however, studies isolated the fungus from humans who had direct contact with infected cats and dogs [5, 17]. High rates of dermatophytosis were also reported earlier by other studies; for instance, 55.5% in cats in Kolkata, India [18], 62.42% in dogs in Baghdad, Iraq [19], 52.2% in humans in Tripoli, Libya [20], and 44.1% in humans in Turkey [21]. The high prevalence rates of the disease reflected its zoonotic nature and confirmed the significant transmission of dermatophytosis in both animals and humans, suggesting the high potentiality of the zoonotic transmission of the disease from cats and dogs to humans.

Multivariable conditions associated with the prevalence of the disease among the examined animals and humans were considered in this study. Animals below the age of 1 year were more susceptible to the infection; perhaps, this might be due to the naivety of their immune systems or skin injuries resulting from sibling or cat socialization [22]. While among humans, the increased rates at younger ages could be due to direct contact and socialization with infected pet cats and dogs since younger people socialize more with cats and dogs than older people.

The prevalence of the disease was also higher in males than in females; however, no statistically significant association was found between infection and sex in both cats and dogs. These findings are in line with those reported earlier by Cafarchia *et al*. [23] and Debnath *et al*. [24]. Thus, cats and dogs of both sexes are considered important sources of human infection. Whereas, this study found that the prevalence of the disease was higher in females than in males (although the difference was not statistically significant). The higher rates in females might be due to the direct contact between infected pets and their households, as females within the area interact with pets more than males. A higher prevalence of the disease in females than in males was reported in earlier studies [25, 26].

In terms of residence, an indoor cat was more susceptible to dermatophytes than an outdoor cat, probably due to the potential leading role of the feline species in the environmental spread of *M. canis*. Infected cats appear to cause substantial environmental contamination. Whereas, dogs seem to be of less importance in the spread of *M. canis* because although they contaminated surfaces, they never contaminated the air [27]. In contrast, the prevalence of dermatophytes was higher among outdoor dogs or dogs reared in shelters than in household dogs; however, the difference was not statistically significant. Such obtained data are in line with those reported by Ibrahim *et al*. [5], Yamada *et al*. [28]. Unsanitary conditions and fungal spore cross-contamination between healthy animals and infected ones confirm the natural route of dermatophyte infections, particularly *M. canis* infections [19].

The prevalence of the disease was also higher among patients who were in contact with infected pets. These findings indicate that direct contact with a contaminated environment exposes humans to infection. Therefore, strict hygienic measures should be followed when rearing pets at home, especially cats.

Higher rates of dermatophytes were reported in animals and humans with affected skin compared to those with apparently healthy skin, perhaps due to defects in the normal function of intact skin as a barrier for a fungal infection that facilitates fungal invasion. These findings are in parallel with those reported earlier by Paryuni *et al*. [1], which indicates that the clinical course of the infection is accelerated by the presence of skin wounds, scars, or burns [29].

In addition to the aforementioned factors (age, gender, skin condition, and contact with pets), COVID-19 has also been considered as a predisposing factor, and its impact on the prevalence of dermatophytes among infected patients was studied. An association between COVID-19 and dermatophytosis was reported among infected patients. The prevalence of dermatophytosis was almost 2 times higher in people with COVID-19 than in those without a history of COVID-19. The differences could be due to the stress impact of COVID-19 on the host’s immune response to dermatophyte infection. To the best of our knowledge, no study has yet been conducted to investigate the association between COVID-19 and dermatophyte infections; thus, we had nothing to compare our findings to in that aspect. However, a previous study reported that dermatomycosis caused by *Trichophyton rubrum* was exacerbated after COVID-19 vaccination [30]. The author hypothesized that the coronavirus vaccine increased serum levels of IL-6, which, in turn, increased serum ferritin levels, thereby creating an environment that is conducive to the development of fungal infections.

## Conclusion

Taken together, this study revealed that the prevalence of dermatophytes, namely, *M. canis*, was high among cats, dogs, and humans. Indoor and outdoor animals and symptomatic or asymptomatic cats and dogs are important sources of infection in the human population. COVID-19 predisposes humans to dermatophytosis. However, more studies need to be conducted to understand the association between COVID-19 and dermatophytosis in humans.

## Authors’ Contributions

KIJ and NAI: Conceptualization, visualization, methodology, software, validation, formal analysis, investigation, resources, data curation, and writing – review and editing. NAI: Writing – original draft preparation, supervision, and project administration. Both authors have read and approved the final manuscript.
